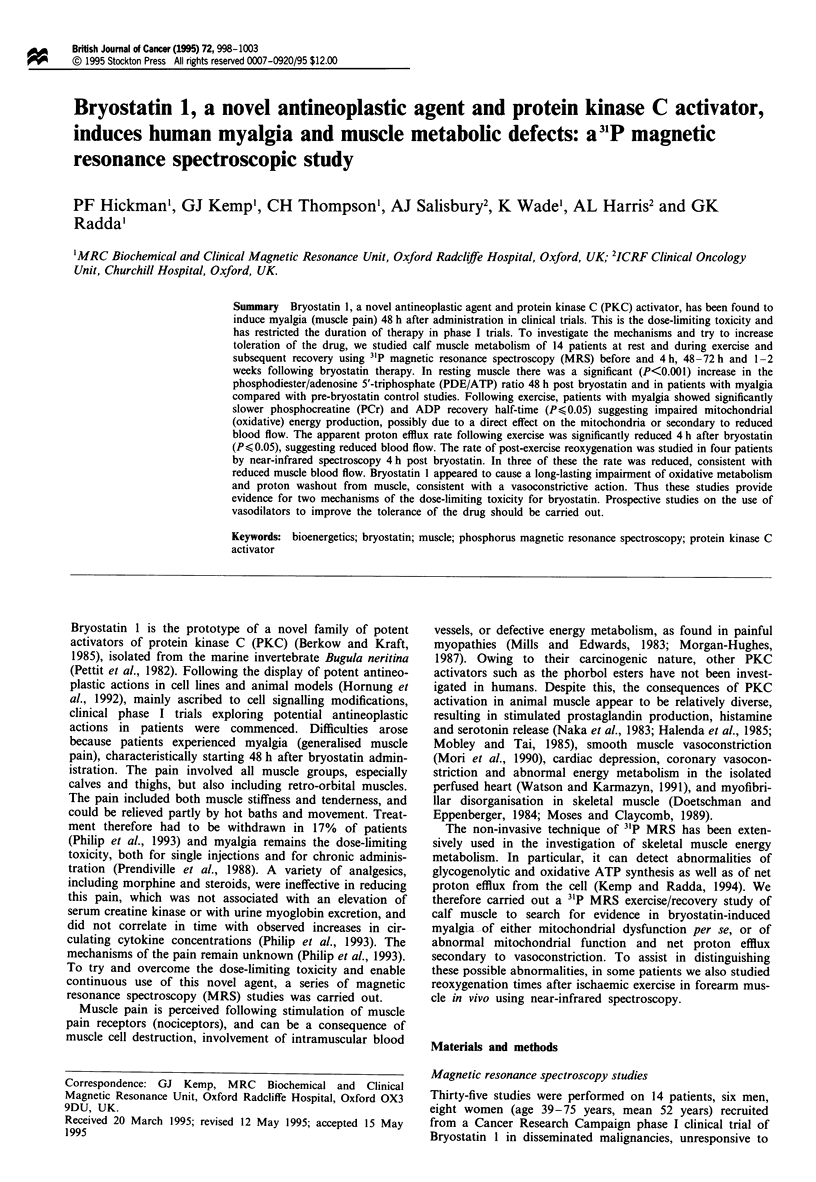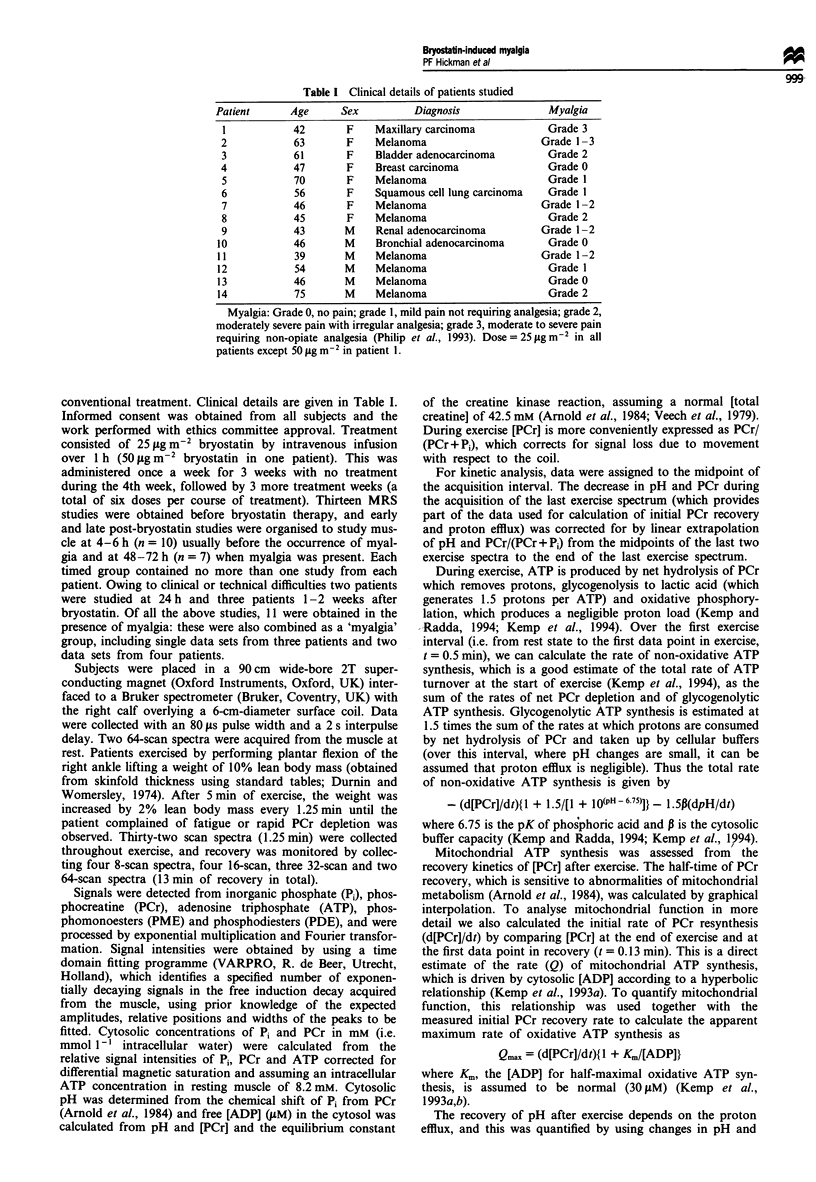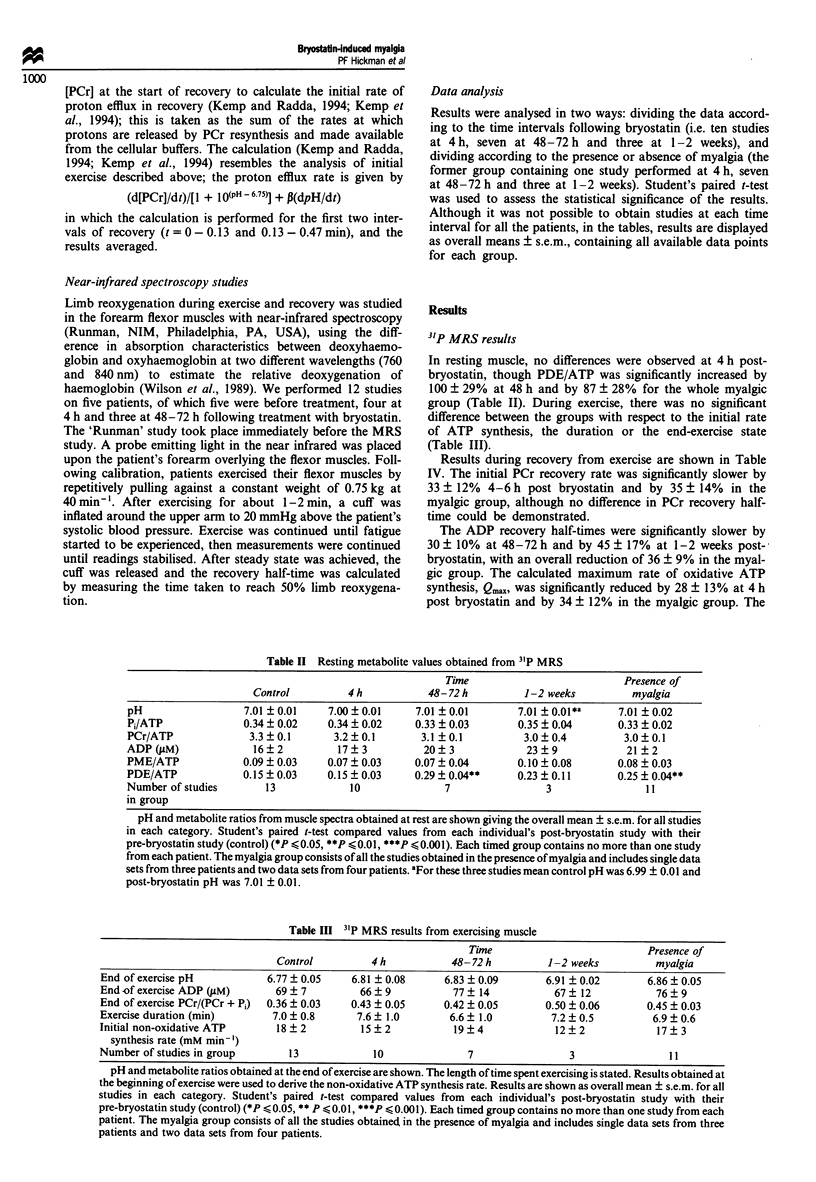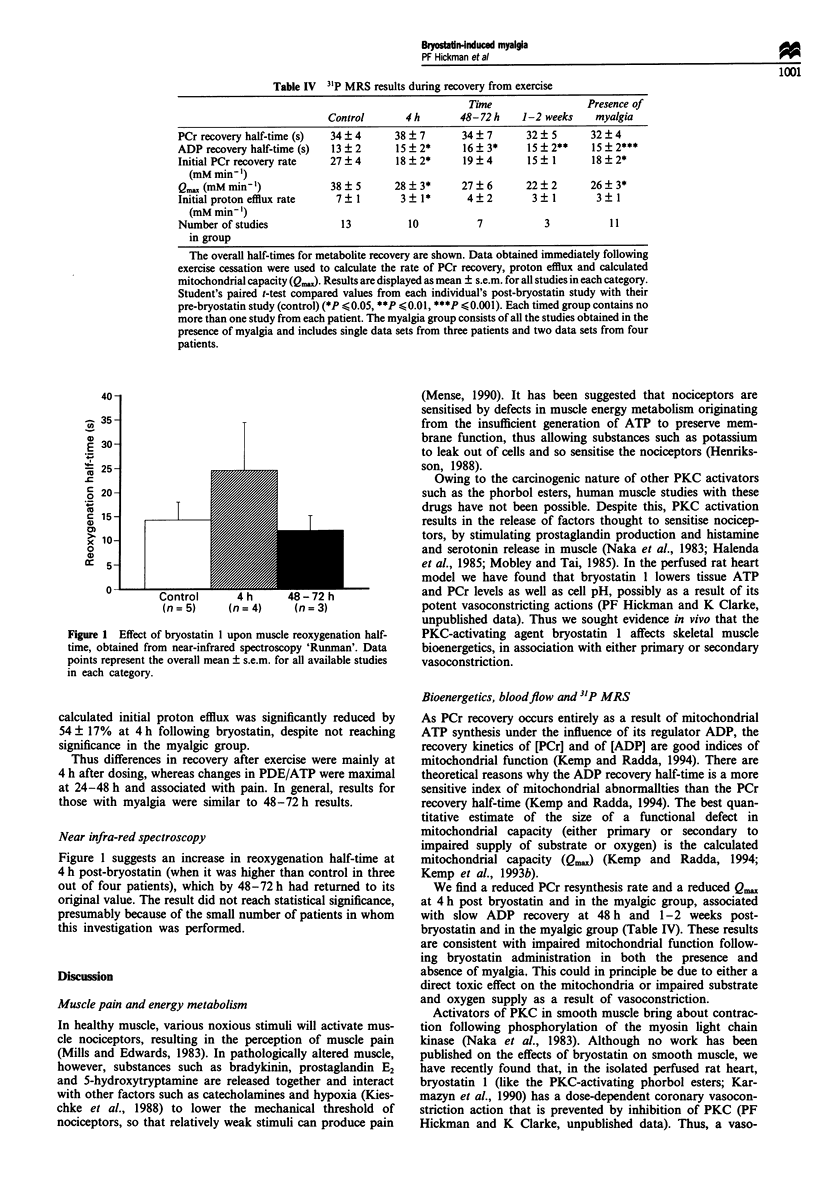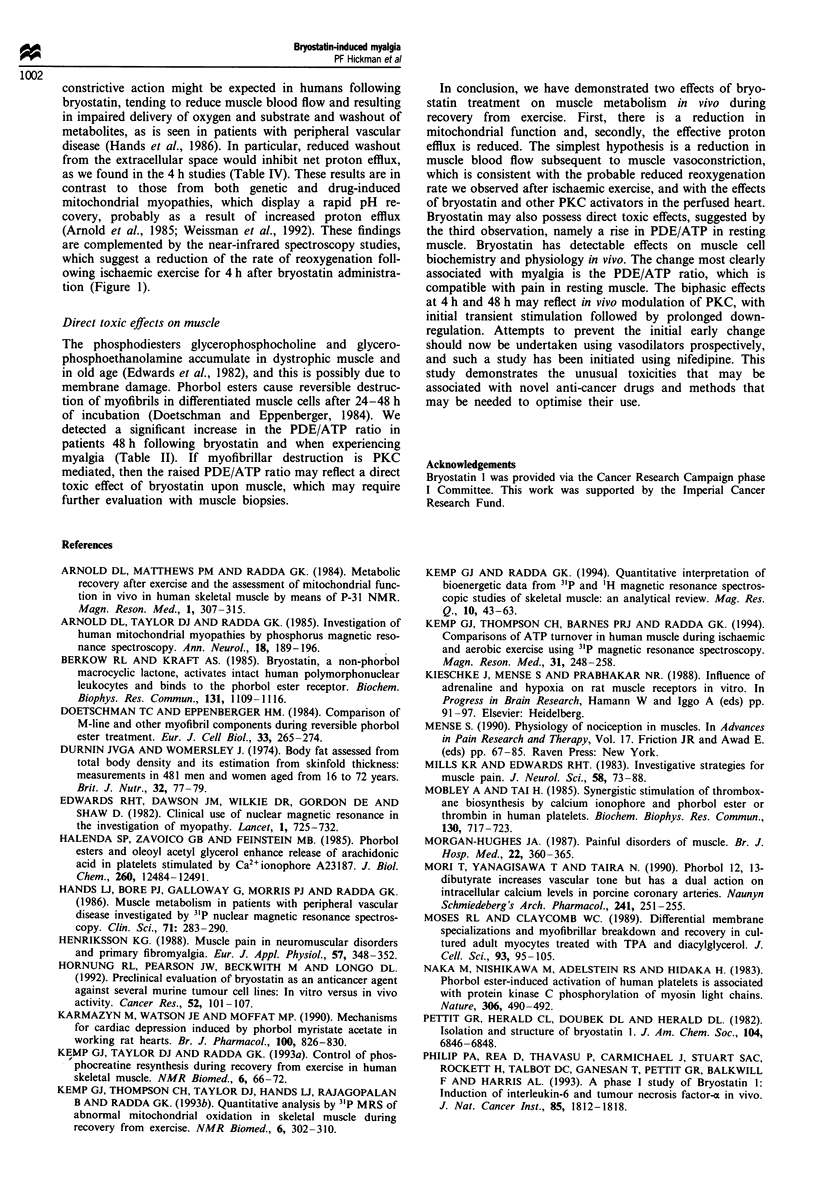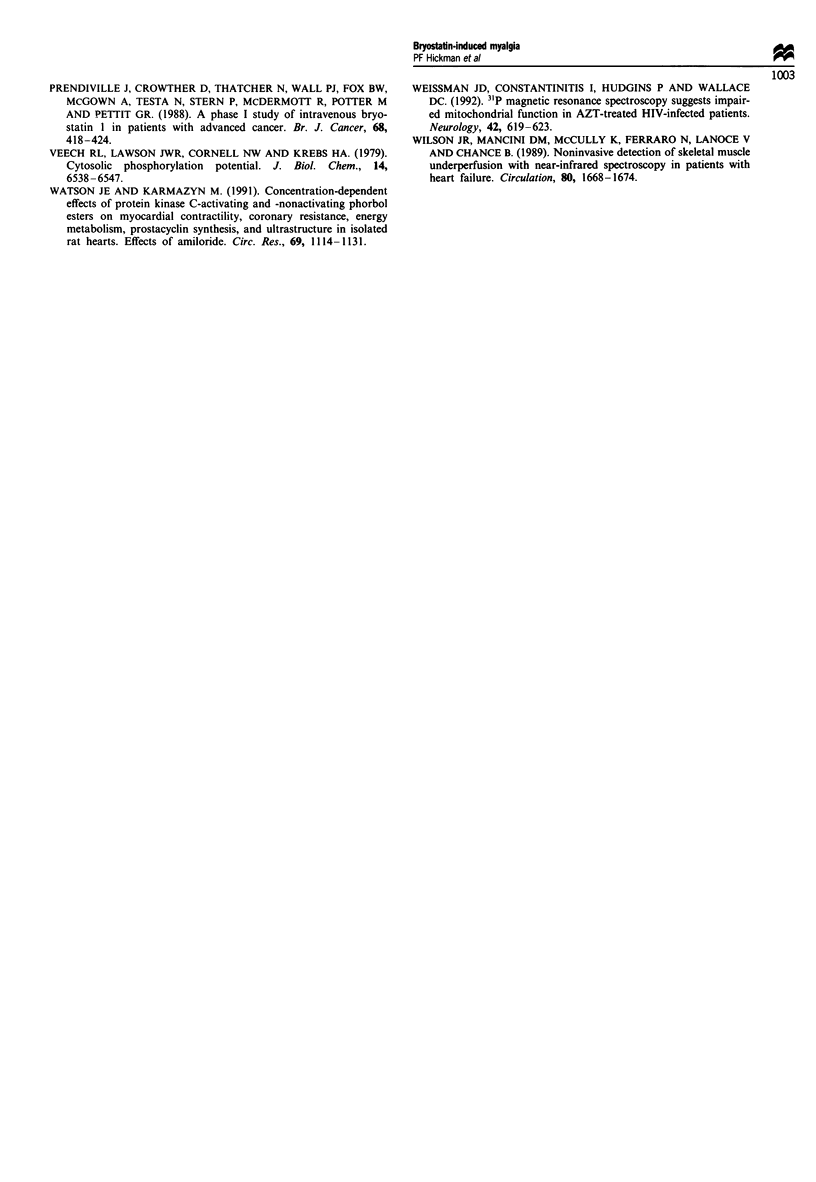# Bryostatin 1, a novel antineoplastic agent and protein kinase C activator, induces human myalgia and muscle metabolic defects: a 31P magnetic resonance spectroscopic study.

**DOI:** 10.1038/bjc.1995.449

**Published:** 1995-10

**Authors:** P. F. Hickman, G. J. Kemp, C. H. Thompson, A. J. Salisbury, K. Wade, A. L. Harris, G. K. Radda

**Affiliations:** MRC Biochemical and Clinical Magnetic Resonance Unit, Oxford Radcliffe Hospital, UK.

## Abstract

Bryostatin 1, a novel antineoplastic agent and protein kinase C (PKC) activator, has been found to induce myalgia (muscle pain) 48 h after administration in clinical trials. This is the dose-limiting toxicity and has restricted the duration of therapy in phase I trials. To investigate the mechanisms and try to increase toleration of the drug, we studied calf muscle metabolism of 14 patients at rest and during exercise and subsequent recovery using 31P magnetic resonance spectroscopy (MRS) before and 4 h, 48-72 h and 1-2 weeks following bryostatin therapy. In resting muscle there was a significant (P < 0.001) increase in the phosphodiester/adenosine 5'-triphosphate (PDE/ATP) ratio 48 h post bryostatin and in patients with myalgia compared with pre-bryostatin control studies. Following exercise, patients with myalgia showed significantly slower phosphocreatine (PCr) and ADP recovery half-time (P < or = 0.05) suggesting impaired mitochondrial (oxidative) energy production, possibly due to a direct effect on the mitochondria or secondary to reduced blood flow. The apparent proton efflux rate following exercise was significantly reduced 4 h after bryostatin (P < or = 0.05), suggesting reduced blood flow. The rate of post-exercise reoxygenation was studied in four patients by near-infrared spectroscopy 4 h post bryostatin. In three of these the rate was reduced, consistent with reduced muscle blood flow. Bryostatin 1 appeared to cause a long-lasting impairment of oxidative metabolism and proton washout from muscle, consistent with a vasoconstrictive action. Thus these studies provide evidence for two mechanisms of the dose-limiting toxicity for bryostatin. Prospective studies on the use of vasodilators to improve the tolerance of the drug should be carried out.


British Journal of Cancer (1995) 72, 998-1003
© 1995 Stockton Press All rights reserved 0007-0920/95 $12.00

### Bryostatin 1, a novel antineoplastic agent and protein kinase C activator, induces human myalgia and muscle metabolic defects: a $^{31}P$ magnetic resonance spectroscopic study

PF Hickman[1], GJ Kemp[1], CH Thompson[1], AJ Salisbury[2], K Wade[1], AL Harris[2] and GK Radda[1]

[1]*MRC Biochemical and Clinical Magnetic Resonance Unit, Oxford Radcliffe Hospital, Oxford, UK;* [2]*ICRF Clinical Oncology Unit, Churchill Hospital, Oxford, UK.*

**Summary** Bryostatin 1, a novel antineoplastic agent and protein kinase C (PKC) activator, has been found to induce myalgia (muscle pain) 48 h after administration in clinical trials. This is the dose-limiting toxicity and has restricted the duration of therapy in phase I trials. To investigate the mechanisms and try to increase toleration of the drug, we studied calf muscle metabolism of 14 patients at rest and during exercise and subsequent recovery using $^{31}P$ magnetic resonance spectroscopy (MRS) before and 4 h, 48-72 h and 1-2 weeks following bryostatin therapy. In resting muscle there was a significant ($P<0.001$) increase in the phosphodiester/adenosine 5′-triphosphate (PDE/ATP) ratio 48 h post bryostatin and in patients with myalgia compared with pre-bryostatin control studies. Following exercise, patients with myalgia showed significantly slower phosphocreatine (PCr) and ADP recovery half-time ($P\leqslant0.05$) suggesting impaired mitochondrial (oxidative) energy production, possibly due to a direct effect on the mitochondria or secondary to reduced blood flow. The apparent proton efflux rate following exercise was significantly reduced 4 h after bryostatin ($P\leqslant0.05$), suggesting reduced blood flow. The rate of post-exercise reoxygenation was studied in four patients by near-infrared spectroscopy 4 h post bryostatin. In three of these the rate was reduced, consistent with reduced muscle blood flow. Bryostatin 1 appeared to cause a long-lasting impairment of oxidative metabolism and proton washout from muscle, consistent with a vasoconstrictive action. Thus these studies provide evidence for two mechanisms of the dose-limiting toxicity for bryostatin. Prospective studies on the use of vasodilators to improve the tolerance of the drug should be carried out.

**Keywords:** bioenergetics; bryostatin; muscle; phosphorus magnetic resonance spectroscopy; protein kinase C activator

Bryostatin 1 is the prototype of a novel family of potent activators of protein kinase C (PKC) (Berkow and Kraft, 1985), isolated from the marine invertebrate *Bugula neritina* (Pettit *et al.*, 1982). Following the display of potent antineoplastic actions in cell lines and animal models (Hornung *et al.*, 1992), mainly ascribed to cell signalling modifications, clinical phase I trials exploring potential antineoplastic actions in patients were commenced. Difficulties arose because patients experienced myalgia (generalised muscle pain), characteristically starting 48 h after bryostatin administration. The pain involved all muscle groups, especially calves and thighs, but also including retro-orbital muscles. The pain included both muscle stiffness and tenderness, and could be relieved partly by hot baths and movement. Treatment therefore had to be withdrawn in 17% of patients (Philip *et al.*, 1993) and myalgia remains the dose-limiting toxicity, both for single injections and for chronic administration (Prendiville *et al.*, 1988). A variety of analgesics, including morphine and steroids, were ineffective in reducing this pain, which was not associated with an elevation of serum creatine kinase or with urine myoglobin excretion, and did not correlate in time with observed increases in circulating cytokine concentrations (Philip *et al.*, 1993). The mechanisms of the pain remain unknown (Philip *et al.*, 1993). To try and overcome the dose-limiting toxicity and enable continuous use of this novel agent, a series of magnetic resonance spectroscopy (MRS) studies was carried out.

Muscle pain is perceived following stimulation of muscle pain receptors (nociceptors), and can be a consequence of muscle cell destruction, involvement of intramuscular blood vessels, or defective energy metabolism, as found in painful myopathies (Mills and Edwards, 1983; Morgan-Hughes, 1987). Owing to their carcinogenic nature, other PKC activators such as the phorbol esters have not been investigated in humans. Despite this, the consequences of PKC activation in animal muscle appear to be relatively diverse, resulting in stimulated prostaglandin production, histamine and serotonin release (Naka *et al.*, 1983; Halenda *et al.*, 1985; Mobley and Tai, 1985), smooth muscle vasoconstriction (Mori *et al.*, 1990), cardiac depression, coronary vasoconstriction and abnormal energy metabolism in the isolated perfused heart (Watson and Karmazyn, 1991), and myofibrillar disorganisation in skeletal muscle (Doetschman and Eppenberger, 1984; Moses and Claycomb, 1989).

The non-invasive technique of $^{31}P$ MRS has been extensively used in the investigation of skeletal muscle energy metabolism. In particular, it can detect abnormalities of glycogenolytic and oxidative ATP synthesis as well as of net proton efflux from the cell (Kemp and Radda, 1994). We therefore carried out a $^{31}P$ MRS exercise/recovery study of calf muscle to search for evidence in bryostatin-induced myalgia of either mitochondrial dysfunction *per se*, or of abnormal mitochondrial function and net proton efflux secondary to vasoconstriction. To assist in distinguishing these possible abnormalities, in some patients we also studied reoxygenation times after ischaemic exercise in forearm muscle *in vivo* using near-infrared spectroscopy.

### Materials and methods

### *Magnetic resonance spectroscopy studies*

Thirty-five studies were performed on 14 patients, six men, eight women (age 39-75 years, mean 52 years) recruited from a Cancer Research Campaign phase I clinical trial of Bryostatin 1 in disseminated malignancies, unresponsive to

Correspondence: GJ Kemp, MRC Biochemical and Clinical Magnetic Resonance Unit, Oxford Radcliffe Hospital, Oxford OX3 9DU, UK.
Received 20 March 1995; revised 12 May 1995; accepted 15 May 1995

Table I Clinical details of patients studied

| Patient | Age | Sex | Diagnosis | Myalgia |
|---|---|---|---|---|
| 1 | 42 | F | Maxillary carcinoma | Grade 3 |
| 2 | 63 | F | Melanoma | Grade 1-3 |
| 3 | 61 | F | Bladder adenocarcinoma | Grade 2 |
| 4 | 47 | F | Breast carcinoma | Grade 0 |
| 5 | 70 | F | Melanoma | Grade 1 |
| 6 | 56 | F | Squamous cell lung carcinoma | Grade 1 |
| 7 | 46 | F | Melanoma | Grade 1-2 |
| 8 | 45 | F | Melanoma | Grade 2 |
| 9 | 43 | M | Renal adenocarcinoma | Grade 1-2 |
| 10 | 46 | M | Bronchial adenocarcinoma | Grade 0 |
| 11 | 39 | M | Melanoma | Grade 1-2 |
| 12 | 54 | M | Melanoma | Grade 1 |
| 13 | 46 | M | Melanoma | Grade 0 |
| 14 | 75 | M | Melanoma | Grade 2 |

Myalgia: Grade 0, no pain; grade 1, mild pain not requiring analgesia; grade 2, moderately severe pain with irregular analgesia; grade 3, moderate to severe pain requiring non-opiate analgesia (Philip *et al.*, 1993). Dose = 25 $\mu g\ m^{-2}$ in all patients except 50 $\mu g\ m^{-2}$ in patient 1.

conventional treatment. Clinical details are given in Table I. Informed consent was obtained from all subjects and the work performed with ethics committee approval. Treatment consisted of 25 $\mu g\ m^{-2}$ bryostatin by intravenous infusion over 1 h (50 $\mu g\ m^{-2}$ bryostatin in one patient). This was administered once a week for 3 weeks with no treatment during the 4th week, followed by 3 more treatment weeks (a total of six doses per course of treatment). Thirteen MRS studies were obtained before bryostatin therapy, and early and late post-bryostatin studies were organised to study muscle at 4-6 h ($n = 10$) usually before the occurrence of myalgia and at 48-72 h ($n = 7$) when myalgia was present. Each timed group contained no more than one study from each patient. Owing to clinical or technical difficulties two patients were studied at 24 h and three patients 1-2 weeks after bryostatin. Of all the above studies, 11 were obtained in the presence of myalgia: these were also combined as a 'myalgia' group, including single data sets from three patients and two data sets from four patients.

Subjects were placed in a 90 cm wide-bore 2T superconducting magnet (Oxford Instruments, Oxford, UK) interfaced to a Bruker spectrometer (Bruker, Coventry, UK) with the right calf overlying a 6-cm-diameter surface coil. Data were collected with an 80 $\mu s$ pulse width and a 2 s interpulse delay. Two 64-scan spectra were acquired from the muscle at rest. Patients exercised by performing plantar flexion of the right ankle lifting a weight of 10% lean body mass (obtained from skinfold thickness using standard tables; Durnin and Womersley, 1974). After 5 min of exercise, the weight was increased by 2% lean body mass every 1.25 min until the patient complained of fatigue or rapid PCr depletion was observed. Thirty-two scan spectra (1.25 min) were collected throughout exercise, and recovery was monitored by collecting four 8-scan spectra, four 16-scan, three 32-scan and two 64-scan spectra (13 min of recovery in total).

Signals were detected from inorganic phosphate ($P_i$), phosphocreatine (PCr), adenosine triphosphate (ATP), phosphomonoesters (PME) and phosphodiesters (PDE), and were processed by exponential multiplication and Fourier transformation. Signal intensities were obtained by using a time domain fitting programme (VARPRO, R. de Beer, Utrecht, Holland), which identifies a specified number of exponentially decaying signals in the free induction decay acquired from the muscle, using prior knowledge of the expected amplitudes, relative positions and widths of the peaks to be fitted. Cytosolic concentrations of $P_i$ and PCr in mM (i.e. mmol $l^{-1}$ intracellular water) were calculated from the relative signal intensities of $P_i$, PCr and ATP corrected for differential magnetic saturation and assuming an intracellular ATP concentration in resting muscle of 8.2 mM. Cytosolic pH was determined from the chemical shift of $P_i$ from PCr (Arnold *et al.*, 1984) and free [ADP] ($\mu$M) in the cytosol was calculated from pH and [PCr] and the equilibrium constant of the creatine kinase reaction, assuming a normal [total creatine] of 42.5 mM (Arnold *et al.*, 1984; Veech *et al.*, 1979). During exercise [PCr] is more conveniently expressed as PCr/(PCr + $P_i$), which corrects for signal loss due to movement with respect to the coil.

For kinetic analysis, data were assigned to the midpoint of the acquisition interval. The decrease in pH and PCr during the acquisition of the last exercise spectrum (which provides part of the data used for calculation of initial PCr recovery and proton efflux) was corrected for by linear extrapolation of pH and PCr/(PCr + $P_i$) from the midpoints of the last two exercise spectra to the end of the last exercise spectrum.

During exercise, ATP is produced by net hydrolysis of PCr which removes protons, glycogenolysis to lactic acid (which generates 1.5 protons per ATP) and oxidative phosphorylation, which produces a negligible proton load (Kemp and Radda, 1994; Kemp *et al.*, 1994). Over the first exercise interval (i.e. from rest state to the first data point in exercise, $t = 0.5$ min), we can calculate the rate of non-oxidative ATP synthesis, which is a good estimate of the total rate of ATP turnover at the start of exercise (Kemp *et al.*, 1994), as the sum of the rates of net PCr depletion and of glycogenolytic ATP synthesis. Glycogenolytic ATP synthesis is estimated at 1.5 times the sum of the rates at which protons are consumed by net hydrolysis of PCr and taken up by cellular buffers (over this interval, where pH changes are small, it can be assumed that proton efflux is negligible). Thus the total rate of non-oxidative ATP synthesis is given by

$$-(d[PCr]/dt)\{1 + 1.5/[1 + 10^{(pH - 6.75)}]\} - 1.5\beta(dpH/dt)$$

where 6.75 is the p$K$ of phosphoric acid and $\beta$ is the cytosolic buffer capacity (Kemp and Radda, 1994; Kemp *et al.*, 1994).

Mitochondrial ATP synthesis was assessed from the recovery kinetics of [PCr] after exercise. The half-time of PCr recovery, which is sensitive to abnormalities of mitochondrial metabolism (Arnold *et al.*, 1984), was calculated by graphical interpolation. To analyse mitochondrial function in more detail we also calculated the initial rate of PCr resynthesis (d[PCr]/d$t$) by comparing [PCr] at the end of exercise and at the first data point in recovery ($t = 0.13$ min). This is a direct estimate of the rate ($Q$) of mitochondrial ATP synthesis, which is driven by cytosolic [ADP] according to a hyperbolic relationship (Kemp *et al.*, 1993*a*). To quantify mitochondrial function, this relationship was used together with the measured initial PCr recovery rate to calculate the apparent maximum rate of oxidative ATP synthesis as

$$Q_{max} = (d[PCr]/dt)\{1 + K_m/[ADP]\}$$

where $K_m$, the [ADP] for half-maximal oxidative ATP synthesis, is assumed to be normal (30 $\mu$M) (Kemp *et al.*, 1993*a*,*b*).

The recovery of pH after exercise depends on the proton efflux, and this was quantified by using changes in pH and

[PCr] at the start of recovery to calculate the initial rate of proton efflux in recovery (Kemp and Radda, 1994; Kemp et al., 1994); this is taken as the sum of the rates at which protons are released by PCr resynthesis and made available from the cellular buffers. The calculation (Kemp and Radda, 1994; Kemp et al., 1994) resembles the analysis of initial exercise described above; the proton efflux rate is given by

$$(d[PCr]/dt)/[1 + 10^{(pH-6.75)}] + \beta(dpH/dt)$$

in which the calculation is performed for the first two intervals of recovery ($t = 0 - 0.13$ and $0.13 - 0.47$ min), and the results averaged.

### Near-infrared spectroscopy studies

Limb reoxygenation during exercise and recovery was studied in the forearm flexor muscles with near-infrared spectroscopy (Runman, NIM, Philadelphia, PA, USA), using the difference in absorption characteristics between deoxyhaemoglobin and oxyhaemoglobin at two different wavelengths (760 and 840 nm) to estimate the relative deoxygenation of haemoglobin (Wilson et al., 1989). We performed 12 studies on five patients, of which five were before treatment, four at 4 h and three at 48–72 h following treatment with bryostatin. The 'Runman' study took place immediately before the MRS study. A probe emitting light in the near infrared was placed upon the patient's forearm overlying the flexor muscles. Following calibration, patients exercised their flexor muscles by repetitively pulling against a constant weight of 0.75 kg at 40 $min^{-1}$. After exercising for about 1–2 min, a cuff was inflated around the upper arm to 20 mmHg above the patient's systolic blood pressure. Exercise was continued until fatigue started to be experienced, then measurements were continued until readings stabilised. After steady state was achieved, the cuff was released and the recovery half-time was calculated by measuring the time taken to reach 50% limb reoxygenation.

### Data analysis

Results were analysed in two ways: dividing the data according to the time intervals following bryostatin (i.e. ten studies at 4 h, seven at 48–72 h and three at 1–2 weeks), and dividing according to the presence or absence of myalgia (the former group containing one study performed at 4 h, seven at 48–72 h and three at 1–2 weeks). Student's paired t-test was used to assess the statistical significance of the results. Although it was not possible to obtain studies at each time interval for all the patients, in the tables, results are displayed as overall means ± s.e.m., containing all available data points for each group.

### Results

### $^{31}P$ MRS results

In resting muscle, no differences were observed at 4 h post-bryostatin, though PDE/ATP was significantly increased by 100 ± 29% at 48 h and by 87 ± 28% for the whole myalgic group (Table II). During exercise, there was no significant difference between the groups with respect to the initial rate of ATP synthesis, the duration or the end-exercise state (Table III).

Results during recovery from exercise are shown in Table IV. The initial PCr recovery rate was significantly slower by 33 ± 12% 4–6 h post bryostatin and by 35 ± 14% in the myalgic group, although no difference in PCr recovery half-time could be demonstrated.

The ADP recovery half-times were significantly slower by 30 ± 10% at 48–72 h and by 45 ± 17% at 1–2 weeks post-bryostatin, with an overall reduction of 36 ± 9% in the myalgic group. The calculated maximum rate of oxidative ATP synthesis, $Q_{max}$, was significantly reduced by 28 ± 13% at 4 h post bryostatin and by 34 ± 12% in the myalgic group. The

**Table II** Resting metabolite values obtained from $^{31}P$ MRS

| | Control | Time: 4 h | Time: 48–72 h | Time: 1–2 weeks | Presence of myalgia |
|---|---|---|---|---|---|
| pH | 7.01 ± 0.01 | 7.00 ± 0.01 | 7.01 ± 0.01 | 7.01 ± 0.01*[a] | 7.01 ± 0.02 |
| $P_i$/ATP | 0.34 ± 0.02 | 0.34 ± 0.02 | 0.33 ± 0.03 | 0.35 ± 0.04 | 0.33 ± 0.02 |
| PCr/ATP | 3.3 ± 0.1 | 3.2 ± 0.1 | 3.1 ± 0.1 | 3.0 ± 0.4 | 3.0 ± 0.1 |
| ADP (μM) | 16 ± 2 | 17 ± 3 | 20 ± 3 | 23 ± 9 | 21 ± 2 |
| PME/ATP | 0.09 ± 0.03 | 0.07 ± 0.03 | 0.07 ± 0.04 | 0.10 ± 0.08 | 0.08 ± 0.03 |
| PDE/ATP | 0.15 ± 0.03 | 0.15 ± 0.03 | 0.29 ± 0.04** | 0.23 ± 0.11 | 0.25 ± 0.04** |
| Number of studies in group | 13 | 10 | 7 | 3 | 11 |

pH and metabolite ratios from muscle spectra obtained at rest are shown giving the overall mean ± s.e.m. for all studies in each category. Student's paired t-test compared values from each individual's post-bryostatin study with their pre-bryostatin study (control) (*$P \leq 0.05$, **$P \leq 0.01$, ***$P \leq 0.001$). Each timed group contains no more than one study from each patient. The myalgia group consists of all the studies obtained in the presence of myalgia and includes single data sets from three patients and two data sets from four patients. [a]For these three studies mean control pH was 6.99 ± 0.01 and post-bryostatin pH was 7.01 ± 0.01.

**Table III** $^{31}P$ MRS results from exercising muscle

| | Control | Time: 4 h | Time: 48–72 h | Time: 1–2 weeks | Presence of myalgia |
|---|---|---|---|---|---|
| End of exercise pH | 6.77 ± 0.05 | 6.81 ± 0.08 | 6.83 ± 0.09 | 6.91 ± 0.02 | 6.86 ± 0.05 |
| End of exercise ADP (μM) | 69 ± 7 | 66 ± 9 | 77 ± 14 | 67 ± 12 | 76 ± 9 |
| End of exercise PCr/(PCr + $P_i$) | 0.36 ± 0.03 | 0.43 ± 0.05 | 0.42 ± 0.05 | 0.50 ± 0.06 | 0.45 ± 0.03 |
| Exercise duration (min) | 7.0 ± 0.8 | 7.6 ± 1.0 | 6.6 ± 1.0 | 7.2 ± 0.5 | 6.9 ± 0.6 |
| Initial non-oxidative ATP synthesis rate (mM $min^{-1}$) | 18 ± 2 | 15 ± 2 | 19 ± 4 | 12 ± 2 | 17 ± 3 |
| Number of studies in group | 13 | 10 | 7 | 3 | 11 |

pH and metabolite ratios obtained at the end of exercise are shown. The length of time spent exercising is stated. Results obtained at the beginning of exercise were used to derive the non-oxidative ATP synthesis rate. Results are shown as overall mean ± s.e.m. for all studies in each category. Student's paired t-test compared values from each individual's post-bryostatin study with their pre-bryostatin study (control) (*$P \leq 0.05$, ** $P \leq 0.01$, ***$P \leq 0.001$). Each timed group contains no more than one study from each patient. The myalgia group consists of all the studies obtained in the presence of myalgia and includes single data sets from three patients and two data sets from four patients.

**Table IV** $^{31}P$ MRS results during recovery from exercise

| | Control | 4 h | *Time* 48–72 h | 1–2 weeks | Presence of myalgia |
|---|---|---|---|---|---|
| PCr recovery half-time (s) | 34 ± 4 | 38 ± 7 | 34 ± 7 | 32 ± 5 | 32 ± 4 |
| ADP recovery half-time (s) | 13 ± 2 | 15 ± 2* | 16 ± 3* | 15 ± 2** | 15 ± 2*** |
| Initial PCr recovery rate (mM min$^{-1}$) | 27 ± 4 | 18 ± 2* | 19 ± 4 | 15 ± 1 | 18 ± 2* |
| $Q_{max}$ (mM min$^{-1}$) | 38 ± 5 | 28 ± 3* | 27 ± 6 | 22 ± 2 | 26 ± 3* |
| Initial proton efflux rate (mM min$^{-1}$) | 7 ± 1 | 3 ± 1* | 4 ± 2 | 3 ± 1 | 3 ± 1 |
| Number of studies in group | 13 | 10 | 7 | 3 | 11 |

The overall half-times for metabolite recovery are shown. Data obtained immediately following exercise cessation were used to calculate the rate of PCr recovery, proton efflux and calculated mitochondrial capacity ($Q_{max}$). Results are displayed as mean ± s.e.m. for all studies in each category. Student's paired *t*-test compared values from each individual's post-bryostatin study with their pre-bryostatin study (control) (*$P \leq 0.05$, **$P \leq 0.01$, ***$P \leq 0.001$). Each timed group contains no more than one study from each patient. The myalgia group consists of all the studies obtained in the presence of myalgia and includes single data sets from three patients and two data sets from four patients.

**Figure 1** Effect of bryostatin 1 upon muscle reoxygenation half-time, obtained from near-infrared spectroscopy 'Runman'. Data points represent the overall mean ± s.e.m. for all available studies in each category.

calculated initial proton efflux was significantly reduced by 54 ± 17% at 4 h following bryostatin, despite not reaching significance in the myalgic group.

Thus differences in recovery after exercise were mainly at 4 h after dosing, whereas changes in PDE/ATP were maximal at 24–48 h and associated with pain. In general, results for those with myalgia were similar to 48–72 h results.

### *Near infra-red spectroscopy*

Figure 1 suggests an increase in reoxygenation half-time at 4 h post-bryostatin (when it was higher than control in three out of four patients), which by 48–72 h had returned to its original value. The result did not reach statistical significance, presumably because of the small number of patients in whom this investigation was performed.

### Discussion

### *Muscle pain and energy metabolism*

In healthy muscle, various noxious stimuli will activate muscle nociceptors, resulting in the perception of muscle pain (Mills and Edwards, 1983). In pathologically altered muscle, however, substances such as bradykinin, prostaglandin $E_2$ and 5-hydroxytryptamine are released together and interact with other factors such as catecholamines and hypoxia (Kieschke *et al.*, 1988) to lower the mechanical threshold of nociceptors, so that relatively weak stimuli can produce pain (Mense, 1990). It has been suggested that nociceptors are sensitised by defects in muscle energy metabolism originating from the insufficient generation of ATP to preserve membrane function, thus allowing substances such as potassium to leak out of cells and so sensitise the nociceptors (Henriksson, 1988).

Owing to the carcinogenic nature of other PKC activators such as the phorbol esters, human muscle studies with these drugs have not been possible. Despite this, PKC activation results in the release of factors thought to sensitise nociceptors, by stimulating prostaglandin production and histamine and serotonin release in muscle (Naka *et al.*, 1983; Halenda *et al.*, 1985; Mobley and Tai, 1985). In the perfused rat heart model we have found that bryostatin 1 lowers tissue ATP and PCr levels as well as cell pH, possibly as a result of its potent vasoconstricting actions (PF Hickman and K Clarke, unpublished data). Thus we sought evidence *in vivo* that the PKC-activating agent bryostatin 1 affects skeletal muscle bioenergetics, in association with either primary or secondary vasoconstriction.

### *Bioenergetics, blood flow and $^{31}P$ MRS*

As PCr recovery occurs entirely as a result of mitochondrial ATP synthesis under the influence of its regulator ADP, the recovery kinetics of [PCr] and of [ADP] are good indices of mitochondrial function (Kemp and Radda, 1994). There are theoretical reasons why the ADP recovery half-time is a more sensitive index of mitochondrial abnormallties than the PCr recovery half-time (Kemp and Radda, 1994). The best quantitative estimate of the size of a functional defect in mitochondrial capacity (either primary or secondary to impaired supply of substrate or oxygen) is the calculated mitochondrial capacity ($Q_{max}$) (Kemp and Radda, 1994; Kemp *et al.*, 1993*b*).

We find a reduced PCr resynthesis rate and a reduced $Q_{max}$ at 4 h post bryostatin and in the myalgic group, associated with slow ADP recovery at 48 h and 1–2 weeks post-bryostatin and in the myalgic group (Table IV). These results are consistent with impaired mitochondrial function following bryostatin administration in both the presence and absence of myalgia. This could in principle be due to either a direct toxic effect on the mitochondria or impaired substrate and oxygen supply as a result of vasoconstriction.

Activators of PKC in smooth muscle bring about contraction following phosphorylation of the myosin light chain kinase (Naka *et al.*, 1983). Although no work has been published on the effects of bryostatin on smooth muscle, we have recently found that, in the isolated perfused rat heart, bryostatin 1 (like the PKC-activating phorbol esters; Karmazyn *et al.*, 1990) has a dose-dependent coronary vasoconstriction action that is prevented by inhibition of PKC (PF Hickman and K Clarke, unpublished data). Thus, a vasoconstrictive action might be expected in humans following bryostatin, tending to reduce muscle blood flow and resulting in impaired delivery of oxygen and substrate and washout of metabolites, as is seen in patients with peripheral vascular disease (Hands *et al.*, 1986). In particular, reduced washout from the extracellular space would inhibit net proton efflux, as we found in the 4 h studies (Table IV). These results are in contrast to those from both genetic and drug-induced mitochondrial myopathies, which display a rapid pH recovery, probably as a result of increased proton efflux (Arnold *et al.*, 1985; Weissman *et al.*, 1992). These findings are complemented by the near-infrared spectroscopy studies, which suggest a reduction of the rate of reoxygenation following ischaemic exercise for 4 h after bryostatin administration (Figure 1).

### *Direct toxic effects on muscle*

The phosphodiesters glycerophosphocholine and glycerophosphoethanolamine accumulate in dystrophic muscle and in old age (Edwards *et al.*, 1982), and this is possibly due to membrane damage. Phorbol esters cause reversible destruction of myofibrils in differentiated muscle cells after 24–48 h of incubation (Doetschman and Eppenberger, 1984). We detected a significant increase in the PDE/ATP ratio in patients 48 h following bryostatin and when experiencing myalgia (Table II). If myofibrillar destruction is PKC mediated, then the raised PDE/ATP ratio may reflect a direct toxic effect of bryostatin upon muscle, which may require further evaluation with muscle biopsies.

In conclusion, we have demonstrated two effects of bryostatin treatment on muscle metabolism *in vivo* during recovery from exercise. First, there is a reduction in mitochondrial function and, secondly, the effective proton efflux is reduced. The simplest hypothesis is a reduction in muscle blood flow subsequent to muscle vasoconstriction, which is consistent with the probable reduced reoxygenation rate we observed after ischaemic exercise, and with the effects of bryostatin and other PKC activators in the perfused heart. Bryostatin may also possess direct toxic effects, suggested by the third observation, namely a rise in PDE/ATP in resting muscle. Bryostatin has detectable effects on muscle cell biochemistry and physiology *in vivo*. The change most clearly associated with myalgia is the PDE/ATP ratio, which is compatible with pain in resting muscle. The biphasic effects at 4 h and 48 h may reflect *in vivo* modulation of PKC, with initial transient stimulation followed by prolonged down-regulation. Attempts to prevent the initial early change should now be undertaken using vasodilators prospectively, and such a study has been initiated using nifedipine. This study demonstrates the unusual toxicities that may be associated with novel anti-cancer drugs and methods that may be needed to optimise their use.

**Acknowledgements**
Bryostatin 1 was provided via the Cancer Research Campaign phase I Committee. This work was supported by the Imperial Cancer Research Fund.

### References

ARNOLD DL, MATTHEWS PM AND RADDA GK. (1984). Metabolic recovery after exercise and the assessment of mitochondrial function in vivo in human skeletal muscle by means of P-31 NMR. *Magn. Reson. Med.*, **1**, 307–315.

ARNOLD DL, TAYLOR DJ AND RADDA GK. (1985). Investigation of human mitochondrial myopathies by phosphorus magnetic resonance spectroscopy. *Ann. Neurol.*, **18**, 189–196.

BERKOW RL AND KRAFT AS. (1985). Bryostatin, a non-phorbol macrocyclic lactone, activates intact human polymorphonuclear leukocytes and binds to the phorbol ester receptor. *Biochem. Biophys. Res. Commun.*, **131**, 1109–1116.

DOETSCHMAN TC AND EPPENBERGER HM. (1984). Comparison of M-line and other myofibril components during reversible phorbol ester treatment. *Eur. J. Cell Biol.*, **33**, 265–274.

DURNIN JVGA AND WOMERSLEY J. (1974). Body fat assessed from total body density and its estimation from skinfold thickness: measurements in 481 men and women aged from 16 to 72 years. *Brit. J. Nutr.*, **32**, 77–79.

EDWARDS RHT, DAWSON JM, WILKIE DR, GORDON DE AND SHAW D. (1982). Clinical use of nuclear magnetic resonance in the investigation of myopathy. *Lancet*, **1**, 725–732.

HALENDA SP, ZAVOICO GB AND FEINSTEIN MB. (1985). Phorbol esters and oleoyl acetyl glycerol enhance release of arachidonic acid in platelets stimulated by $Ca^{2+}$ionophore A23187. *J. Biol. Chem.*, **260**, 12484–12491.

HANDS LJ, BORE PJ, GALLOWAY G, MORRIS PJ AND RADDA GK. (1986). Muscle metabolism in patients with peripheral vascular disease investigated by $^{31}P$ nuclear magnetic resonance spectroscopy. *Clin. Sci.*, **71**: 283–290.

HENRIKSSON KG. (1988). Muscle pain in neuromuscular disorders and primary fibromyalgia. *Eur. J. Appl. Physiol.*, **57**, 348–352.

HORNUNG RL, PEARSON JW, BECKWITH M AND LONGO DL. (1992). Preclinical evaluation of bryostatin as an anticancer agent against several murine tumour cell lines: In vitro versus in vivo activity. *Cancer Res.*, **52**, 101–107.

KARMAZYN M, WATSON JE AND MOFFAT MP. (1990). Mechanisms for cardiac depression induced by phorbol myristate acetate in working rat hearts. *Br. J. Pharmacol.*, **100**, 826–830.

KEMP GJ, TAYLOR DJ AND RADDA GK. (1993*a*). Control of phosphocreatine resynthesis during recovery from exercise in human skeletal muscle. *NMR Biomed.*, **6**, 66–72.

KEMP GJ, THOMPSON CH, TAYLOR DJ, HANDS LJ, RAJAGOPALAN B AND RADDA GK. (1993*b*). Quantitative analysis by $^{31}P$ MRS of abnormal mitochondrial oxidation in skeletal muscle during recovery from exercise. *NMR Biomed.*, **6**, 302–310.

KEMP GJ AND RADDA GK. (1994). Quantitative interpretation of bioenergetic data from $^{31}P$ and $^{1}H$ magnetic resonance spectroscopic studies of skeletal muscle: an analytical review. *Mag. Res. Q.*, **10**, 43–63.

KEMP GJ, THOMPSON CH, BARNES PRJ AND RADDA GK. (1994). Comparisons of ATP turnover in human muscle during ischaemic and aerobic exercise using $^{31}P$ magnetic resonance spectroscopy. *Magn. Reson. Med.*, **31**, 248–258.

KIESCHKE J, MENSE S AND PRABHAKAR NR. (1988). Influence of adrenaline and hypoxia on rat muscle receptors in vitro. In *Progress in Brain Research*, Hamann W and Iggo A (eds) pp. 91–97. Elsevier: Heidelberg.

MENSE S. (1990). Physiology of nociception in muscles. In *Advances in Pain Research and Therapy*, Vol. 17. Friction JR and Awad E. (eds) pp. 67–85. Raven Press: New York.

MILLS KR AND EDWARDS RHT. (1983). Investigative strategies for muscle pain. *J. Neurol. Sci.*, **58**, 73–88.

MOBLEY A AND TAI H. (1985). Synergistic stimulation of thromboxane biosynthesis by calcium ionophore and phorbol ester or thrombin in human platelets. *Biochem. Biophys. Res. Commun.*, **130**, 717–723.

MORGAN-HUGHES JA. (1987). Painful disorders of muscle. *Br. J. Hosp. Med.*, **22**, 360–365.

MORI T, YANAGISAWA T AND TAIRA N. (1990). Phorbol 12, 13-dibutyrate increases vascular tone but has a dual action on intracellular calcium levels in porcine coronary arteries. *Naunyn Schmiedeberg's Arch. Pharmacol.*, **241**, 251–255.

MOSES RL AND CLAYCOMB WC. (1989). Differential membrane specializations and myofibrillar breakdown and recovery in cultured adult myocytes treated with TPA and diacylglycerol. *J. Cell. Sci.*, **93**, 95–105.

NAKA M, NISHIKAWA M, ADELSTEIN RS AND HIDAKA H. (1983). Phorbol ester-induced activation of human platelets is associated with protein kinase C phosphorylation of myosin light chains. *Nature*, **306**, 490–492.

PETTIT GR, HERALD CL, DOUBEK DL AND HERALD DL. (1982). Isolation and structure of bryostatin 1. *J. Am. Chem. Soc.*, **104**, 6846–6848.

PHILIP PA, REA D, THAVASU P, CARMICHAEL J, STUART SAC, ROCKETT H, TALBOT DC, GANESAN T, PETTIT GR, BALKWILL F AND HARRIS AL. (1993). A phase I study of Bryostatin 1: Induction of interleukin-6 and tumour necrosis factor-α in vivo. *J. Nat. Cancer Inst.*, **85**, 1812–1818.

PRENDIVILLE J, CROWTHER D, THATCHER N, WALL PJ, FOX BW, MCGOWN A, TESTA N, STERN P, MCDERMOTT R, POTTER M AND PETTIT GR. (1988). A phase I study of intravenous bryostatin 1 in patients with advanced cancer. *Br. J. Cancer*, **68**, 418-424.

VEECH RL, LAWSON JWR, CORNELL NW AND KREBS HA. (1979). Cytosolic phosphorylation potential. *J. Biol. Chem.*, **14**, 6538-6547.

WATSON JE AND KARMAZYN M. (1991). Concentration-dependent effects of protein kinase C-activating and -nonactivating phorbol esters on myocardial contractility, coronary resistance, energy metabolism, prostacyclin synthesis, and ultrastructure in isolated rat hearts. Effects of amiloride. *Circ. Res.*, **69**, 1114-1131.

WEISSMAN JD, CONSTANTINITIS I, HUDGINS P AND WALLACE DC. (1992). $^{31}$P magnetic resonance spectroscopy suggests impaired mitochondrial function in AZT-treated HIV-infected patients. *Neurology*, **42**, 619-623.

WILSON JR, MANCINI DM, MCCULLY K, FERRARO N, LANOCE V AND CHANCE B. (1989). Noninvasive detection of skeletal muscle underperfusion with near-infrared spectroscopy in patients with heart failure. *Circulation*, **80**, 1668-1674.